# Partnering to Strengthen Health Systems and Improve Access to Quality Cancer Care

**DOI:** 10.1200/GO.22.00148

**Published:** 2022-10-17

**Authors:** Amy Israel

**Affiliations:** 1 Novartis, East Hanover, NJ

Access within health care is a universal concern, independent of country income levels or the percentage of gross domestic product investment in health. Addressing the ongoing COVID-19 pandemic has shed light on the hidden access challenges across systems that present barriers to care for many people. These include cultural and language barriers, diminished trust in the health system, and lack of familiarity with negotiating care.^1^ Although innovation has addressed certain barriers such as eliminating up-front costs during the pandemic, we still see immense hurdles to care.^1^ In addition to participation barriers, staffing constraints, fragmentation of care, and uneven distribution of resources can pose significant obstacles.^2,3^ A recent commentary in *Communications Medicine* emphasized that access to cancer care is dependent on ensuring the presence of *staff, stuff, space, systems, and social support*,^3^ a concept coined by one of the leading architects of improving access, Paul Farmer.

For example, screening and diagnosis initiatives for breast cancer are critical to identify cancers in early stages when there is more opportunity to improve outcomes and at lower cost to the health system.^4,5^ However, screening programs can only succeed if subsequent diagnosis is confirmed and treatment is initiated within appropriate timelines.^4,6^ This cannot occur if the system lacks the capacity in terms of resources, staffing, knowledge, or diagnostic capabilities to confirm diagnosis and refer the patient for care. Another challenge occurs when efforts to address access are implemented with a focus on speed and quantity rather than on quality of care. The *Lancet Commission on High Quality Health in the SDG Era* has warned that poor quality care is now a bigger barrier to reducing mortality in low- and middle-income countries than insufficient access.^7^

At Novartis, we are committed to lowering system barriers that prevent patients from accessing treatment, and we believe that health systems strengthening is most impactful when multiple stakeholders bring their unique strengths to the table to collaborate on solutions. In 2020, we launched our One Novartis Health Systems Strengthening Framework for teams to systematically identify a critical barrier within the cascade of care preventing access, and, together with health systems players, cocreate a solution to lower that barrier. We held an internal challenge called Breaking Barriers to support Novartis country teams in collaborating with their health systems on solutions to these obstacles. The eligibility criteria were simple, focusing on three key areas for cancer care: precision oncology, care integration, and data in action. Teams were required to address an issue that was acknowledged by their health system partner(s) as a genuine barrier to care. Despite our concern that local teams and health systems might be hesitant to embark on this journey amid a global health emergency, we received more than 80 proposals from 45 countries in a short period of time. We observed recurrent themes across geographies or country income level:

1. Delayed or inadequate screening and diagnosis because of:
  a) Lack of patient access to local and timely diagnostic services
  b) Lack of provider knowledge or capacity to trigger testing
  c) Lack of genetic/molecular diagnostic capacity and/or reimbursement
  d) The need for greater patient navigation support during diagnosis and treatment initiation
2. Delays and backlogs in diagnosis or treatment because of bottlenecks in the cancer care system, often exacerbated by COVID-19
3. Deficiencies in use of data to drive decision making (eg, lack of translation of histopathological results into registries and lack of infrastructure and consensus to collect real-world evidence)

None of these themes will surprise the global oncology community, especially given the alarms that have been sounded over the need to catch up from COVID-19 backlogs and global shortage of pathology professionals and pathology infrastructure. However, somewhat surprising to us was the remarkable consensus on the types of solutions coidentified by the teams and their health system partners:

Restructuring of care continuum to strengthen patient navigation and increase efficiency:

- Digital pathway mapping tools
- Use of advanced technologies to drive efficiencies
- Digital platforms to increase clinic-level efficiencies and reduce screening backlogs

Improving data collection and use:

- Building and strengthening registries and biobanks
- Supporting use of digital tools, such as artificial intelligence, to leverage data in diagnosis and treatment decisions

There has not only been much discussion in health policy literature over the past two years of the potential for digital tools and advanced analytics to bridge gaps in health care but also skepticism as to whether systems are ready for such a shift. It is clear, at least from an oncology perspective, that they are ready; they are eager to test innovative solutions and understand how they can deliver value.

In selecting proposals for the internal challenge, we prioritized scalability, collaboration, and potential impact for patients. Although initiatives will range across country income levels, we are highlighting three of the early starters which reflect common challenges referenced above. The initiative in Ireland aims to address diagnostic delays because of capacity bottlenecks in laboratory services across the country. They will start by validating the use of liquid biopsies in advanced non-small cell lung cancer to shorten turnaround time. This is being done in alignment with a national working group to establish new pathology pathways. In the second initiative, in Portugal, an artificial intelligence–driven hospital capacity simulation tool is being trialed to identify pain points in the system and run scenario testing to evaluate the impact of potential resource optimization solutions without compromising quality of care. Third, in Poland, a pan-system approach to link care across levels will use a machine learning platform to support identification of patients at high risk for rare blood diseases at the primary care level; these more robust data can then be used for diagnosis and decision making at the specialist level. As part of the pan-system approach, a patient-held tool to scan blood test results to aid in screening for hematologic diseases and a digital resource will upskill pathologists on recognition of rare blood cancers, enabling faster and more accurate diagnosis, and strengthening the data repository to improve algorithms in the future. While partnering in all three initiatives, it is important to note that the data remain in the health system and are not shared with Novartis.

The intention of Breaking Barriers was to create and test sustainable interventions which could solve for identified critical obstacles in the care cascade. Once robustly analyzed for effectiveness, the health system actors can take the evidence-based practices and scale for improved health service delivery. In the above described initiatives, the health system barrier was identified or validated through consultation with local health system representatives. All partners are active collaborators throughout the process to adapt for feasibility and drive toward sustainability.

It is no coincidence that the most easily scalable initiatives make use of advanced digital technologies that are easily transferrable, adaptable to different facilities and locations, and require little supporting infrastructure. Inclusion of the technology sector is key for accessing creative solutions that challenge the status quo and problem solving along the way. Most of these interventions offer suggestions for leapfrogging solutions in low-resource settings. The health systems we are working with have welcomed the opportunity to trial these digital solutions to address identified gaps; they are not meant to impose an extraneous service on the system. These three initiatives are at the proof-of-concept stage. As yet, there are not sufficient data to determine if they offer solutions to the barriers identified or what modifications would be necessary to enable their scalability to other settings. In particular, we have to consider the relevance and feasibility of replicating an initiative from a high-income country in a lower-income setting, but we can test adapted versions of these approaches. For instance, the liquid biopsy validation project is intended to address the shortage of pathology services and speed time to an initial molecular assessment that can then be validated through tissue biopsy. Clearly, this only provides a solution if the infrastructure exists for follow-up. This is why we emphasize local barrier validation and solution creation and the importance of cross-sectoral collaboration. These are all necessary elements to deliver the different pieces of the puzzle.

Too often, we are cowed by the enormity of macrolevel access challenges. The problem is too complex, too widespread, and too expensive to fix. We took a different approach and asked country teams to take a micro perspective, partnering on discrete problems that could be mitigated through creative solutions. Although the tools and approaches differ in each of these efforts, the common thread is direct benefit to patients through potentially faster diagnosis, timely initiation of treatment, and data-informed treatment decisions. Even initiatives that may not deliver expected results will benefit patients by supporting the system to learn what does and does not work. And already we are seeing that the simple act of initiating discussion around these barriers and potential solutions triggers an interesting dialogue among stakeholders to drive change. In parallel, internally at Novartis this has triggered compelling dialogue on how we as a pharmaceutical company in a highly regulated environment can contribute to such change. In planning to submit this commentary, we—perhaps naively—thought that we would have troves of impact data on these initiatives at this time. This is clearly not the case, although we do look forward to reporting on outcomes and greater details of each initiative at a later date. One of our key learnings on this journey is that this is not easy. Planning, alignment (internal and external), and true cocreating take time.

Good collaborations are based on a foundation of trust. Although this is a pillar of the Novartis approach, it cannot be taken for granted and must be earned. Again, this takes time. We are committed to continuing on this path, learning as we go, and reducing one barrier at a time.
