# Erratum

**DOI:** 10.1200/GO.22.00399

**Published:** 2022-12-16

The article by Israel entitled “Partnering to Strengthen Health Systems and Improve Access to Quality Cancer Care” (*JCO Glob Oncol* 10.1200/GO.22.00148) was published online October 17, 2022, with an error.

The contact information in the online author list and corresponding author section read as:

CORRESPONDING AUTHOR Amy Israel, MSW, 1 Health Plaza, East Hanover, NJ 07936; e-mail: amy.israel@novartis.com.

It should have read as:

CORRESPONDING AUTHOR Amy Israel, MSW, 1 Health Plaza, East Hanover, NJ 07936; e-mail: amy.israel@icloud.com.

This has been corrected as of December 16, 2022. The author apologizes for the error.

